# Iatrogenic cerebral amyloid angiopathy: two new cases and systematic review of case reports with neuropathological data

**DOI:** 10.1186/s42466-025-00423-x

**Published:** 2025-09-03

**Authors:** Ana Sofia Costa, João Pinho, Arno Reich, Omid Nikoubashman, Kay Nolte, Joachim Weis, Christian Boy, Felix M. Mottaghy, Jörg B. Schulz, Kathrin Reetz

**Affiliations:** 1 Department of Neurology, University Hospital RWTH Aachen, Pauwelsstr. 30, 52074 Aachen, Germany; 2 JARA Brain Institute Molecular Neuroscience and Neuroimaging, Research Centre Jülich and RWTH Aachen University, Aachen, Germany; 3 Department of Diagnostic and Interventional Neuroradiology, University Hospital RWTH Aachen, Aachen, Germany; 4 Institute of Neuropathology, University Hospital RWTH Aachen, Aachen, Germany; 5 Department of Nuclear Medicine, University Hospital RWTH Aachen, Aachen, Germany

**Keywords:** Cerebral amyloid angiopathy, Dementia, Intracerebral hemorrhage, Neuropathology

## Abstract

Awareness concerning iatrogenic cerebral amyloid angiopathy (iCAA) is increasing but its pathophysiology remains unclear. We discuss the implications of the clinical, imaging and neuropathological findings in two previously unpublished cases of probable iCAA: a 55-year-old female presenting with rapidly progressive cognitive impairment, showing imaging and histological evidence of CAA and having undergone neurosurgical treatment at the age of 6; and a 56-year-old male with a four-year history of recurring intracerebral hemorrhages (ICH) and neurosurgical intervention at the age of 5. In the first case, a brain biopsy was performed.

Additionally, a systematic review of iCAA cases with neuropathological data suggests that most of the patients have concomitant amyloid parenchymal deposition and none or minimal tau pathology. The pathophysiological role of tau pathology in iCAA remains unclear. iCAA patients presenting with cognitive impairment without symptomatic ICH may be underdiagnosed.

**Supplementary Information:**

The online version contains supplementary material available at 10.1186/s42466-025-00423-x.

## Introduction

Cerebral amyloid angiopathy (CAA) manifesting decades after neurosurgical procedures involving the utilization of cadaveric material, such as dura mater, is gaining increasing awareness [1], and diagnostic criteria for iatrogenic CAA (iCAA) were recently proposed [2] (Additional File 1). Current understanding of the pathophysiology of iCAA suggests a prion-like transmission of amyloid-β seeds from cadaveric material or neurosurgical instruments to the brain. Many patients with iCAA had had traumatic brain injury in the past, and its potential causal role is difficult to disentangle [1–4]. We report two previously unpublished cases of iCAA, perform a systematic review of iCAA cases with neuropathological data and discuss the implications of the findings.

### Case 1

**Fig. 1:**
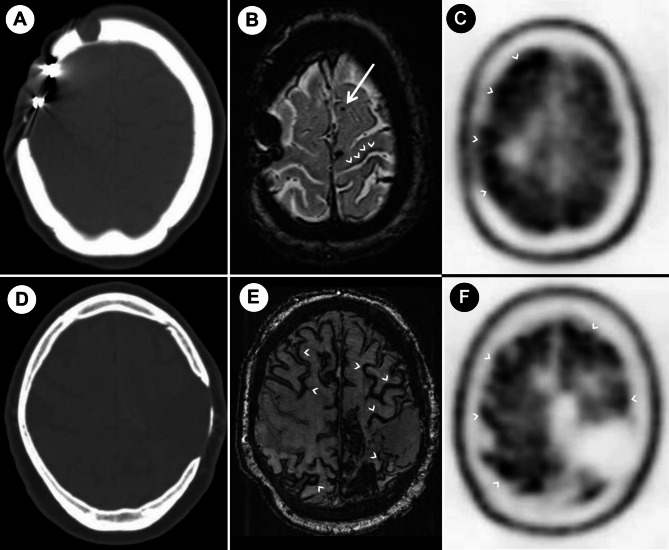
Patient 1: computed tomography showing right-sided fronto-parietal cranial bone defect related to neurosurgical treatment (**A**); susceptibility weighted magnetic resonance imaging showing cortical microbleeds (arrow) and cortical superficial siderosis (arrowheads) (**B**); amyloid 18 F-flutemetamol-PET showing a diffuse right-side predominant (arrowheads) supratentorial cortical uptake (**C**). Patient 2: computed tomography showing left-sided fronto-parietal cranial bone defect related to neurosurgical treatment (**D**); susceptibility weighted magnetic resonance imaging showing severe cortical superficial siderosis (arrowheads) (**E**); amyloid 18 F-flutemetamol-PET showing diffuse supratentorial cortical uptake (arrowheads) (**F**)

**Fig. 2:**
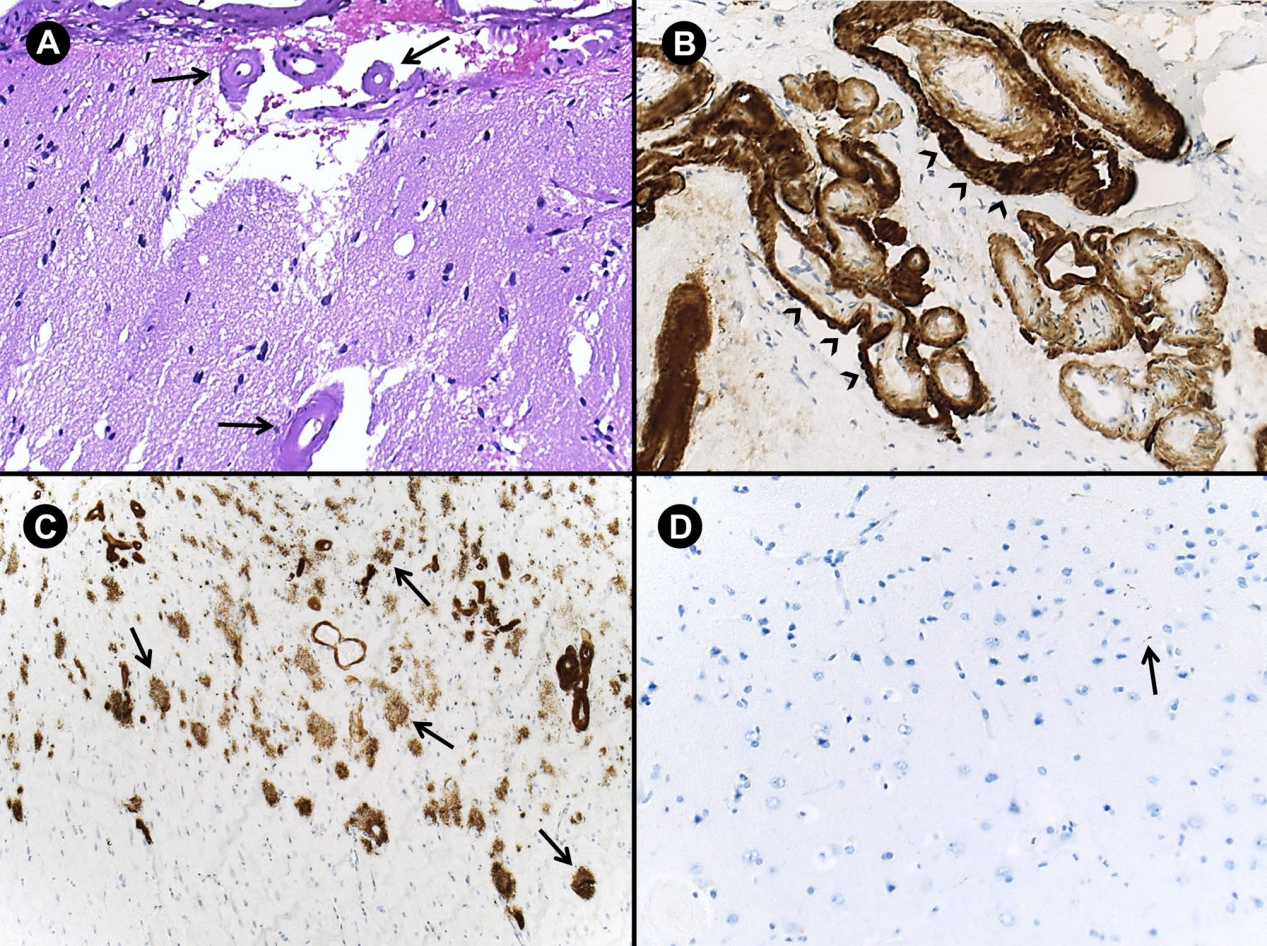
Neuropathological findings in Patient 1: eosinophilic wall thickening of leptomeningeal and cortical arteries (arrows) without inflammatory infiltrates (**A**, hematoxylin-eosin staining); extensive amyloid-β deposition in cortical arteries (arrowheads) (**B**, immunostaining for amyloid-β with monoclonal mouse anti-human beta-amyloid); extensive amyloid-β-positive diffuse plaques (arrows) (**C**, immunostaining for amyloid-β with monoclonal mouse anti-human beta-amyloid); scarce tau pathology with rare neuropil threads (arrow) (**D**, immunostaining for tau protein with AT8 monoclonal antibody)

A 55-year-old female developed a rapidly progressive cognitive impairment with memory, spatial orientation and language complaints, with relevant functional impact on daily living activities. When she was 6 years old, she had a severe neurological disease interpreted as meningoencephalitis and underwent neurosurgical treatment with right-sided fronto-parietal craniectomy (surgical records unavailable). No comorbidities were known and familial history for dementia or stroke was negative. Six months after onset, she presented with a Montreal Cognitive Assessment of 16/30 and Mini Mental State Examination of 23/30. A comprehensive neuropsychological examination revealed mild dementia with impairments in attention, executive functions, visuo-perceptive processing, language and episodic memory. MRI showed chronic parenchymal defects related to meningoencephalitis, multiple lobar microbleeds and cortical superficial siderosis (cSS) (Fig. 1B). Amyloid 18 F-flutemetamol-PET showed a diffuse, right-side predominant, supratentorial cortical uptake (Fig. 1C). CSF neurodegeneration markers revealed low amyloid-β-42 (256 pg/mL), low-normal amyloid-β42/40 ratio (0.52) and normal phospho-tau (57 pg/mL). Genetic testing for APP, PSEN1 and PSEN2 mutations was negative. APOE genotype was E3/E2. The clinical presentation of a rapidly progressive young-onset dementia with neuroimaging evidence of microbleeds raised the possibility of a vasculitis of the CNS with involvement of the small vessels. Thus, a right-sided frontal lobe biopsy was performed to exclude vasculitis and other diagnoses such as active immune-mediated or infectious encephalitis, intravascular angiocentric lymphoma, hemorrhagic micrometastases and neuronal intranuclear inclusion disease. Histopathology revealed eosinophilic arterial wall thickening, fragmentation of the elastic lamina with double-barrel appearance, extensive amyloid-β deposition in leptomeningeal and cortical arterioles (Fig. 2A-B), and amyloid-β-positive diffuse plaques (Fig. 2C). There were no identifiable neuritic plaques and tau pathology was limited to scarce neuropil threads (Fig. 2D). There were no signs of inflammation or microglial activation.

### Case 2

A 56-year-old male had a first left parietal intracerebral hemorrhage (ICH) when he was 52 years old. During the following four years he had four recurrent lobar ICHs (left frontal with acute convexity subarachnoid hemorrhage, right occipital, left parietal, and left frontal with acute convexity subarachnoid hemorrhage). When he was 5 years old, he suffered a severe traumatic brain injury and underwent neurosurgical treatment with left fronto-parietal craniectomy (surgical records unavailable). He had no known comorbidities. His mother was diagnosed with late onset Alzheimer´s disease, otherwise familial history for dementia or stroke was negative. Three years after the first ICH, the patient presented a severe aphasia, a left-sided homonymous hemianopsia and bilateral apraxia. Apart from impairment in complex visual processing and language-dependent deficits, comprehensive neuropsychological examination showed no additional cognitive deficits. MRI revealed a post-traumatic parenchymal defect, extensive cSS and a single lobar microbleed (Fig. 1E). Amyloid 18 F-flutemetamol-PET revealed diffuse supratentorial cortical uptake, predominantly in frontoparietal regions (Fig. 1F). CSF neurodegeneration markers (11 days after the second ICH) revealed a low amyloid-β-42 (424 pg/mL), normal amyloid-β42/40 ratio (1.0), increased total-tau (598 pg/mL), mildly increased phospho-tau (69 pg/mL). Genetic testing for APP, PSEN1 and PSEN2 mutations was negative. APOE genotype was E3/E3. Brain biopsy was declined by the patient at the time of the third ICH.

### Systematic review

We performed a search in MEDLINE using the search term (“iatrogenic” AND “cerebral amyloid angiopathy”) from inception until 31.05.2025 and retrieved 66 results. Using backward citation search we identified 12 additional relevant reports. We retrieved and reviewed the full texts of the 78 reports and identified original reports of cases diagnosed with iatrogenic CAA (*n* = 39 reports). Among these papers, 26 reports provided neuropathological data. We excluded one report for describing cases already previously reported. From the reports we identified 38 patients with iCAA with neuropathological data, and we also included our “case 1” in the final analysis (Additional File 2). Mean age at first presentation was 39 years, 65% of patients were male, and mean time between potential exposure and first presentation was 35 years. ICH was the first manifestation in 26/32 patients. Neuropathological samples were obtained by performing biopsy in 31 patients, and 8 patients were autopsied. In all patients there was evidence of vascular amyloid deposition, the majority of reports specified leptomeningeal and/or cortical vessel deposition of amyloid (*n* = 23). Presence of amyloid cerebral parenchymal deposits was reported in 21 patients, mostly in form of “diffuse plaques” or “diffuse deposits”. Only 1 patient was reported to have no amyloid cerebral parenchymal deposits and in 17 patients this was not mentioned. Presence of cerebral tau pathology was reported in 13 patients (neuritic plaques = 9; neurofibrillary tangles = 8; neuropil threads = 6), and reported as minimal or rare in 6 patients. Seven patients were reported to have no tau pathology, whereas in 19 patients this was not mentioned.

## Discussion

These two cases depict classical cognitive and hemorrhagic manifestations of CAA occurring five decades after cerebral neurosurgical procedures with presumable dura mater reconstruction in the early 1970s. We did not have access to surgical records, and it remains unknown if cadaveric dura mater was used in these two patients. However, the two cases fulfil diagnostic criteria for probable iCAA [2] (Additional File 1). There is a predominance of ICH presentation in the published case series of iCAA [2–5], and patients presenting with cognitive impairment, transient focal neurological episodes or epileptic seizures may be underdiagnosed. Although ICH location and the site of initial surgery appear to spatially collocate, imaging findings in CAA are not restricted to the location of the initial injury or surgery [3, 5].

The profile of CSF neurodegeneration markers and positive amyloid-PET scans in these two patients reflect the presence of amyloid pathology, which was described in previous studies [6, 7]. The neuropathological findings in the patient with cognitive presentation confirm extensive vascular amyloid deposition without inflammation and provide evidence to understand pathophysiological mechanisms of iCAA. We found extensive amyloid-β-positive diffuse plaques without significant tau pathology. Our systematic review suggests that concomitant amyloid parenchymal deposition in iCAA is frequent, at least in the reports where it is mentioned. However, tau pathology appears to be absent or minimal in most of the cases in which it was examined. In contrast, relevant tau pathology is present in most patients with neuropathologically-defined moderate-to-severe sporadic CAA (Braak stage V-VI in 65% of patients; moderate-to-severe CERAD neuritic plaque score in 73% of patients) [8]. As has been previously observed, the pathophysiological role of tau pathology in iCAA remains unclear. Supporting previous observations, the lack of significant tau pathology in our first case suggests that contrary to Alzheimer´s disease, tau pathology may not be the driving pathophysiological mechanism of cognitive impairment in iCAA [9].

In the first case, we did not find any perivascular or intramural lympho-histiocytic inflammatory infiltrates in leptomeningeal and cortical arteries, which excluded the presence of CAA-related inflammation or amyloid-β-related angiitis [10]. Although the current diagnostic criteria for iCAA propose that significant inflammation should be absent [2] (Additional File 1), there is some evidence suggesting that inflammatory changes compatible with CAA-related inflammation may also occur in the context of iCAA [11, 12].

Leptomeningeal and cerebral biopsy is not mandatory for the diagnosis of iCAA, however, it may be important in selected cases to exclude differential diagnoses which may have specific treatments [13]. One of the main differential diagnoses in middle-aged patients with rapidly progressive cognitive impairment, white matter lesions, multiple microbleeds and no signs of vasculitis in angiographic studies is primary or secondary small-vessel vasculitis of the central nervous system, which requires histopathological diagnosis [14].

In conclusion, we report detailed clinical, imaging and pathological characteristics of two patients with iCAA who developed symptoms five decades after neurosurgical procedures. Future detailed study of neuropathological data may help to understand pathophysiology of iCAA.

## Supplementary Information

Below is the link to the electronic supplementary material.

Supplementary Material 1

Supplementary Material 2

## Data Availability

Not applicable.

## Abbreviations

CAA: Cerebral amyloid angiopathy
CSF: Cerebrospinal fluid
iCAA: Iatrogenic cerebral amyloid angiopathy
ICH: Intracerebral hemorrhage
MMSE: Mini-mental state examination
MoCA: Montreal cognitive assessment
MRI: Magnetic resonance imaging
PET: Positron emission tomography
