# Supplementary material for: Iatrogenic cerebral amyloid angiopathy: two new cases and systematic review of case reports with neuropathological data
Source: Neurol Res Pract. 2025 Sep 3;7(1):63. doi: 10.1186/s42466-025-00423-x (PMC12409930; doi:10.1186/s42466-025-00423-x)
Supplement: Supplementary file 1 — Supplementary Material 1 [file 42466_2025_423_MOESM1_ESM.docx]

**Additional File 1**. Diagnostic criteria for iatrogenic cerebral amyloid angiopathy (iCAA) proposed by Banerjee and collaborators (2022)

**1. Age of onset**

- Symptom onset before age of 55 years (ie, below the age threshold for ‘probable’ or ‘possible’ CAA within the modified Boston criteria^9^); *strongly suggestive (although note ascertainment bias)*
- *Note: diagnosis cannot be excluded based on age alone, and should be considered in people aged 55 years or above, should they meet the other criteria (detailed below*)

**2. History of potential exposure (≥1)**

- Procedure or treatment using cadaveric human CNS tissues (ie, brain, meninges, pituitary-derived hormones); *strongly suggestive*
- Relevant neurosurgical procedure (ie, those involving the brain, spinal cord, posterior eye)
- *Note: diagnosis can be considered if history of alternative potential exposure and all other criteria are met*

**3. Clinical and radiological features consistent with a diagnosis of CAA**

3.1. Clinical (≥1 of the following features, at presentation or during disease course):

- Intracerebral haemorrhage or convexity subarachnoid haemorrhage (single or multiple)
- Transient focal neurological episodes ('amyloid spells')
- Focal seizures (with or without secondary generalisation)
- Cognitive impairment not attributable to another cause (including acute stroke)

3.2. Radiological (≥1 of the following features):

- CT:
  - Lobar intracerebral haemorrhage
  - Convexity subarachnoid haemorrhage
- MRI (blood sensitive sequences; T2*-GRE, SWI)
  - Cerebral microbleeds with predominantly lobar distribution, distant from sites of parenchymal intracerebral haemorrhage
  - Cortical superficial siderosis (focal or disseminated) on MR blood sensitive sequences

**4. Evidence of amyloid-beta accumulation in the central nervous system**

- Positive amyloid-PET scan
- Supportive CSF features (reductions of Aβ−42, Aβ−40)
- Brain biopsy demonstrating vascular Aβ deposition, in the absence of significant inflammation
- *Notes: A positive amyloid-PET scan in isolation might not necessarily be specific for Aβ accumulation, depending on the tracer used; correlation with either CSF Aβ measures, brain biopsy findings and/or genetic testing for non-Aβ CAAs (details below) is advised; Presence of significant inflammation might support an alternative diagnosis of CAA-related inflammation or Aβ related angiitis (ABRA)*

**5. Exclusion of genetic causes of amyloid-beta central nervous system disease:**

- Duplications of *APP* (including Trisomy 21, where relevant)
- Mutations of *APP*, *PSEN1*, *PSEN2*
- In cases where CNS Aβ deposition has not been confirmed by other means (CSF Aβ measures, brain biopsy), next-generation sequencing for mutations resulting in non-Aβ CAA (*CST3*, *TTR*, *GSN*, *PRNP*, *ITM2B*) should be considered

Probable iCAA: criteria 2, 3, 4 and 5 must be met as a minimum

Possible iCAA: can be considered if criteria 1, 2 and 3 are met
