# Supplementary material for: Iatrogenic cerebral amyloid angiopathy: two new cases and systematic review of case reports with neuropathological data
Source: Neurol Res Pract. 2025 Sep 3;7(1):63. doi: 10.1186/s42466-025-00423-x (PMC12409930; doi:10.1186/s42466-025-00423-x)
Supplement: Supplementary file 2 — Supplementary Material 2 [file 42466_2025_423_MOESM2_ESM.docx]

Additional File 2: Description of iatrogenic cerebral amyloid angiopathy cases with neuropathological data found in the systematic review

| **Reference** | **Year** | **Number of patients** | **Age at potential exposure** | **Age at first manifestation** | **First manifestation** | **Biopsy / Autopsy** | **Vascular amyloid deposition** | **Cerebral parenchymal amyloid pathology** | **Cerebral parenchymal tau pathology** |
| --- | --- | --- | --- | --- | --- | --- | --- | --- | --- |
| 1 | 2012 | 1 | childhood | 38 y | ICH | biopsy | yes | not reported | not reported |
| 2 | 2017 | 1 | 1y | 32y | ICH | biopsy | yes | not reported | not reported |
| 3 | 2018 | 4 | 3y, 1y, 1y, 20y | 33y, 31y, 36y, 57y | ICH, ICH, ICH, ICH | biopsy, biopsy, autopsy, autopsy | yes | yes, no, yes, yes | no, no, yes (“occasional neuropil thread”), yes (CERAD score 1, Braak and Braak stage II) |
| 4 | 2018 | 1 | 2y | 46y | ICH | both | yes | yes | yes (“near absence”) |
| 5 | 2019 | 1 | 1y | 29y | ICH | biopsy | yes | yes | yes (“minimal”) |
| 6 | 2019 | 2 | 11y, 2y | 41y, 27y | generalized tonic-clonic seizures, ICH | biopsy, biopsy | yes | yes, yes | no, no |
| 7 | 2019 | 1 | 7m | 30y | ICH | biopsy | yes | not reported | not reported |
| 8 | 2020 | 1 | 2y | 34y | ICH | biopsy | yes | yes | no |
| 9 | 2021 | 3 | 4y, 3y, 6y | 44y, 39y, 45y | ICH, ICH, cSAH | biopsy, biopsy, biopsy | yes | yes, yes, yes | yes, yes,  yes (“widespread”) |
| 10 | 2021 | 1 | 5y | 34y | ICH | biopsy | yes | yes | not reported |
| 11 | 2021 | 1 | early childhood | 29 | ICH | biopsy | yes | not reported | not reported |
| 12 | 2022 | 2 | 4y, 2y | 37y, 36y | ICH, ICH | biopsy, biopsy | yes | yes, yes | yes (“scattered”), yes (“scattered”) |
| 13 | 2023 | 1 | 6m | 41y | transient focal neurological episodes | biopsy | yes | not reported | not reported |
| 14 | 2023 | 7 | no individual patient data reported | no individual patient data reported | no individual patient data reported | biopsy (n=4); autopsy (n=3) | yes | not reported | not reported |
| 15 | 2023 | 1 | 23y | 56y | ICH | biopsy | yes | not reported | not reported |
| 16 | 2023 | 1 | 2y | 27y | ICH | biopsy | yes | not reported | not reported |
| 17 | 2023 | 1 | 2y | 51y | ICH | biopsy | yes | yes | yes |
| 18 | 2024 | 1 | 2y | 43y | ICH | biopsy | yes | yes | not reported |
| 19 | 2024 | 1 | 10y | 42y | ICH | biopsy | yes | not reported | not reported |
| 20 | 2024 | 1 | 9y | 46y | ICH | autopsy | yes | yes | yes (“rare neuritic plaques”) |
| 21 | 2024 | 1 | 3m | 35y | ICH | biopsy | yes | yes | no |
| 22 | 2025 | 1 | 9y | 42y | ICH | biopsy | yes | yes | no |
| 23 | 2025 | 1 | 9y | 46y | ICH | autopsy | yes | not reported | not reported |
| 24 | 2025 | 1 | 9m | 38y | cognitive impairment | biopsy | yes | yes | yes |
| 25 | 2025 | 1 | 1y | 37y | transient focal neurological episodes | biopsy | yes | not reported | not reported |
| Case 1 | 2025 | 1 | 6y | 55y | cognitive impairment | biopsy | yes | yes | yes (minimal) |

y: years; m: months; ICH: intracerebral hemorrhage; cSAH: convexal subarachnoidal hemorrhage

**REFERENCES**

1. Ehling R, Helbok R, Beer R, Lackner P, Broessner G, Pfausler B, Röcken C, Aguzzi A, Chemelli A, Schmutzhard E. Recurrent intracerebral haemorrhage after coitus: a case report of sporadic cerebral amyloid angiopathy in a younger patient. Eur J Neurol. 2012 Mar;19(3):e29-31.

2. Nakayama Y, Mineharu Y, Arawaka Y, Nishida S, Tsuji H, Miyake H, Yamaguchi M, Minamiguchi S, Takagi Y, Miyamoto S. Cerebral amyloid angiopathy in a young man with a history of traumatic brain injury: a case report and review of the literature. Acta Neurochir (Wien). 2017 Jan;159(1):15-18.

3. Jaunmuktane Z, Quaegebeur A, Taipa R, Viana-Baptista M, Barbosa R, Koriath C, Sciot R, Mead S, Brandner S. Evidence of amyloid-β cerebral amyloid angiopathy transmission through neurosurgery. Acta Neuropathol. 2018 May;135(5):671-679.

4. Hervé D, Porché M, Cabrejo L, Guidoux C, Tournier-Lasserve E, Nicolas G, Adle-Biassette H, Plu I, Chabriat H, Duyckaerts C. Fatal Abeta cerebral amyloid angiopathy 4 decades after a dural graft at the age of 2 years. Acta Neuropathol. 2018 May;135(5):801-803.

5. Giaccone G, Maderna E, Marucci G, Catania M, Erbetta A, Chiapparini L, Indaco A, Caroppo P, Bersano A, Parati E, Di Fede G, Caputi L. Iatrogenic early onset cerebral amyloid angiopathy 30 years after cerebral trauma with neurosurgery: vascular amyloid deposits are made up of both Aβ40 and Aβ42. Acta Neuropathol Commun. 2019 May 2;7(1):70.

6. Banerjee G, Adams ME, Jaunmuktane Z, Alistair Lammie G, Turner B, Wani M, Sawhney IMS, Houlden H, Mead S, Brandner S, Werring DJ. Early onset cerebral amyloid angiopathy following childhood exposure to cadaveric dura. Ann Neurol. 2019 Feb;85(2):284-290.

7. Hamaguchi T, Komatsu J, Sakai K, Noguchi-Shinohara M, Aoki S, Ikeuchi T, Yamada M. Cerebral hemorrhagic stroke associated with cerebral amyloid angiopathy in young adults about 3 decades after neurosurgeries in their infancy. J Neurol Sci. 2019 Apr 15;399:3-5.

8. Raposo N, Planton M, Siegfried A, Calviere L, Payoux P, Albucher JF, Viguier A, Delisle MB, Uro-Coste E, Chollet F, Bonneville F, Olivot JM, Pariente J. Amyloid-β transmission through cardiac surgery using cadaveric dura mater patch. J Neurol Neurosurg Psychiatry. 2020 Apr;91(4):440-441.

9. Jaunmuktane Z, Banerjee G, Paine S, Parry-Jones A, Rudge P, Grieve J, Toma AK, Farmer SF, Mead S, Houlden H, Werring DJ, Brandner S. Alzheimer’s disease neuropathological change three decades after iatrogenic amyloid‑β transmission. Acta Neuropathol. 2021 Jul;142(1):211-215.

10. Yoshiki K, Hirose G, Kumahashi K, Kohda Y, Ido K, Shioya A, Misaki K, Kasuga K. Follow-up study of a patient with early onset cerebral amyloid angiopathy following childhood cadaveric dural graft. Acta Neurochir (Wien). 2021 May;163(5):1451-1455.

11. Clark A, Barpujari A, Lucke-Wold B, Porche K, Laurent D, Koch M, Decker M. Cerebral amyloid angiopathy: early presentation in a patient with prior neurosurgical interventions: Case report. Romanian Neurosurgery 2021;XXXV(4):499-502.

12. Kellie JF, Campbell BCV, Watson R, Praeger AJ, Nair G, Murugasu A, Rowe CC, Masters CL, Collins S, McLean C, Yassi N. Amyloid-beta (Abeta)-Related Cerebral Amyloid Angiopathy Causing Lobar Hemorrhage Decades After Childhood Neurosurgery. Stroke. 2022 Aug;53(8):e369-e374.

13. Sezgin M, Taşdelen S, Ekizoğlu E, Yeşilot N, Çoban O. Iatrogenic cerebral amyloid angiopathy: a rare case beyond diagnostic criteria with 3 years follow-up. Neurol Sci. 2024 Feb;45(2):805-808.

14. Pikija S, Pretnar-Oblak J, Frol S, Malojcic B, Gattringer T, Rak-Frattner K, Staykov D, Salmaggi A, Milani R, Magdic J, Iglseder S, Trinka E, Kraus T, Toma A, DiFrancesco JC, Tabaee Damavandi P, Fabin N, Bersano A, de la Riva Juez P, Albajar Gomez I, Storti B, Fandler-Höfler S. Iatrogenic cerebral amyloid angiopathy: A multinational case series and individual patient data analysis of the literature. Int J Stroke. 2024 Mar;19(3):314-321.

15. Muller C. Case report of iatrogenic cerebral amyloid angiopathy after exposure to Lyodura: an Australian perspective. Front Neurosci. 2023 May 5;17:1185267.

16. Purrucker JC, Röcken C, Reuss D. Iatrogenic cerebral amyloid angiopathy rather than sporadic CAA in younger adults with lobar intracerebral haemorrhage. Amyloid. 2023 Dec;30(4):434-436.

17. Milani R, Mazzeo LA, Vismara D, Salemi I, Dainese E, Maderna E, Pellencin E, Catania M, Campanella N, Di Fede G, Giaccone G, Salmaggi A. Spontaneous intracerebral haemorrhage associated with early-onset cerebral amyloid angiopathy and Alzheimer's disease neuropathological changes five decades after cadaveric dura mater graft. Acta Neuropathol Commun. 2023 Feb 24;11(1):30.

18. Wolf T, Chammas A, Lannes B, Lhermitte B. [Cerebral amyloid angiopathy]. Ann Pathol. 2024 Nov;44(6):486-490.

19. Choi MCY, Law THP, Chen S, Cheung WSK, Yim C, Ng OKS, Au LWC, Mok VCT, Woo PYM. Case Report: Taxifolin for neurosurgery-associated early-onset cerebral amyloid angiopathy. Front Neurol. 2024 Mar 19;15:1360705.

20. Fabjan M, Jurečič A, Jerala M, Oblak JP, Frol S. Recurrent Intracerebral Haematomas Due to Amyloid Angyopathy after Lyodura Transplantation in Childhood. Neurol Int. 2024 Mar 4;16(2):327-333.

21. Jensen-Kondering U, Heß K, Flüh C, Kuhlenbäumer G, Margraf NG. A Rare Case of Iatrogenic Prion-like Pathogenesis of Cerebral Amyloid Angiopathy. Dtsch Arztebl Int. 2024 Jan 26;121(2):68-69.

22. Kawarabayashi T, Nakamura T, Takatama S, Miyamoto N, Iwai T, Naito I, Sugawara T, Ishizawa K, Hashimoto K, Amari M, Ikeuchi T, Kasahara H, Ikeda Y, Takatama M, Shoji M. A case of the iatrogenic transmission of vascular Aß40 amyloid. Amyloid. 2025 Mar;32(1):81-83.

23. Frol S, Zupan M, Pretnar Oblak J, Velnar T, Splavski B. Iatrogenic cerebral amyloid angiopathy: Two cases linked to childhood cadaveric dural transplantation for different intracranial pathologies, diagnosed using the simplified Edinburgh computed tomography criteria. Surg Neurol Int. 2025 May 2;16:165.

24. Courret T, Le Quang M, Renou P, Penchet G, Tourdias T. Rapid Evolution of an Iatrogenic Cerebral Amyloid Angiopathy. Neurology. 2025 Mar 11;104(5):e213402.

25. Hernández-Fernández F, Martínez-Fernández I, Barbella-Aponte R, Vilar IF, Ayo-Martín O, García-García J, Collado R, Andrés A, Hernández-Guillamón M, Pena Pardo FJ, Barrena C, de la Fuente M, Serrano-Heras G, Melero M, Setién EL, López L, Segura T. Iatrogenic cerebral amyloid angiopathy and Alzheimer's disease co-pathology. Ann Clin Transl Neurol. 2025 Jan;12(1):235-241.
